# Annotations

**Published:** 1887-02-12

**Authors:** 


### Annotations.

Sir James Picton on Homœopathy.

"At thirty man suspects himself a fool: knows it at forty, and reforms his plan." At the annual meeting of the subscribers to the Liverpool Homœopathic Dispensaries, Sir James Picton endeavoured to act the part of mediator between what he called "Homœopathy" and "Allopathy." It is a good thing to try to break down needless "walls of partition," which different sections of mankind have from time to time built up between themselves. Undoubtedly many such walls are not only useless but ridiculous. Men who have reached middle age get tired of strife, and are generally anxious to find a *modus vivendi*, whereby, at least, the peace may be preserved between them and their fellows. But (to use a famous phrase) it must be "peace with honour." Before we can accept Sir James Picton's well-meant efforts at reconciliation, we must be sure that there is no sacrifice of truth and honesty required on either side. For our own part—and we say it with all candour—we cannot for our life understand what "Homœopathy" is; neither, for that matter, is "Allopathy" at all more tangible or definable. There is no such thing as either the one or the other. There was originally a *principle* in Homœopathy, which gave some justification to the cognomen—viz., "similia similibus curantur." But now that that principle has been shown by innumerable experiments to be absolutely fallacious, there is no differentiating formula which will enable us to say what Homœopathy is. The truth is, that so-called Homœopaths cut themselves off from the great body of scientific practitioners by a voluntary and useless act of schism. If they were content to be medical men like others, they could practise according to any principle they pleased, and nobody would say a word. But when they hoist the flag of Homœopathy they *ipso facto* declare that Homœopathy is right, and by consequence that everything not Homœopathic is wrong. But that puts other practitioners on the defensive; and they *must* then either become Homœopaths, or say why they do not do so. And that, of course, leads to the denial of the claims of Homœopathy as a principle, and sometimes to the denunciation of its practitioners. If Homœopaths wish to be recognised by other medical men, let them be content to be known simply as medical men, and put away their conspicuous badge of separation, for which there is no justification in any known principle of science or sense.

The Proposal to change Hospital Sunday.

Men in high positions are not always blessed with the possession of sound judgment. If they were, the Bishop of London would hardly have proposed to postpone Hospital Sunday in favour of the Church House scheme. No doubt it may be a good thing to have a church house, but perhaps there is no one, not even the Bishop, who will venture to say that the house is *absolutely indispensable* for any reason. But is there anyone who will deny that the money collected for hospitals on Hospital Sunday is absolutely indispensable? Surely, if we have to make a choice between a comfortable clerical clubhouse on the one hand, and the due provision for the sick and suffering poor on the other, no man of ordinary feeling—not to speak of Christian sympathy—will hesitate for a moment what choice to make. We have nothing to say against the church house. But we must insist that it is a question solely for church people, and perhaps for the richer among them. The Hospital Sunday collection is everybody's affair, and especially does it belong to the poor and sick. We do not know of anything, unless it were some great national calamity, which should for a moment claim precedence over, or even be put into competition with, the Hospital Sunday Fund. The Committee of the Fund have deserved well of the public for the firm and decided stand they have taken in the matter.

Is Medical Practice Humbug?

A gentleman (or a lady) who appends the signature (in the Latin tongue) "Nobody," writes to say, among other things, that vast numbers of people believe nine-tenths of the medical treatment practised to be not only "pure fudge," but "absolutely destructive of human health." "Whether homœopathy is a delusion or not," he adds, "it is perfectly clear that patients thrive and live under it quite as well as under orthodox treatment. If this be so, the ordinary medical practice must be a humbug and worse." We publish this because we think that no man or profession is the worse for being occasionally pulled up, and asked for a reason for his faith and practice. There is just enough of truth in it to give it a certain degree of point. As practised by some legally qualified medical men, no doubt treatment is properly described by the terms "pure fudge," "humbug," etc. But it must be remembered that there are doctors and doctors. We admit that there are "*Jerry*" doctors, just as there are "*Jerry*" builders, and "*Jerry*" parsons, and "*Jerry*" *nobodies.* But we are quite sure that the art of medicine is in itself as honest and straightforward as any other practical art, and as far removed from anything like "fudge," or "humbug," as the art of carpentering, or ploughing, or any other downright and honest work. The right-minded doctor never practises make-believe. He tells people honestly what he believes to be the matter with them; what they should do for their recovery; whether, and when, he thinks they will recover; and how they may avoid the like illness for the future. All this he does as the result of his thoroughly scientific education and honestly gathered experience. Such a man is probably much less of a humbug than "*Nemo.*"

